# First Pharmacokinetic Data of Tenofovir Alafenamide Fumarate and Tenofovir With Dolutegravir or Boosted Protease Inhibitors in African Children: A Substudy of the CHAPAS-4 Trial

**DOI:** 10.1093/cid/ciad267

**Published:** 2023-05-09

**Authors:** Hylke Waalewijn, Alexander J Szubert, Roeland E Wasmann, Lubbe Wiesner, Chishala Chabala, Mutsa Bwakura-Dangarembizi, Shafic Makumbi, Joan Nangiya, Vivian Mumbiro, Veronica Mulenga, Victor Musiime, Lara N Monkiewicz, Anna L Griffiths, Alasdair Bamford, Katja Doerholt, Paolo Denti, David M Burger, Diana M Gibb, Helen M McIlleron, Angela Colbers, for the Children with HIV in Africa – Pharmacokinetics and Acceptability of Simple second-line antiretroviral regimens (CHAPAS-4) Trial Team

**Affiliations:** Department of Pharmacy, Research Institute for Medical Innovation, Radboud University Medical Center, Nijmegen, The Netherlands; Division of Clinical Pharmacology, Department of Medicine, University of Cape Town, South Africa; Medical Research Council Clinical Trials Unit, University College London, United Kingdom; Division of Clinical Pharmacology, Department of Medicine, University of Cape Town, South Africa; Division of Clinical Pharmacology, Department of Medicine, University of Cape Town, South Africa; Department of Paediatrics and Child Health, School of Medicine, University of Zambia; Children’s Hospital, University Teaching Hospital, Lusaka, Zambia; University of Zimbabwe Clinical Research Centre, Harare; Joint Clinical Research Centre, Mbarara Regional Centre of Excellence, Mbarara, Uganda; Joint Clinical Research Centre, Research Department, Kampala, Uganda; University of Zimbabwe Clinical Research Centre, Harare; Children’s Hospital, University Teaching Hospital, Lusaka, Zambia; Joint Clinical Research Centre, Research Department, Kampala, Uganda; Department of Paediatrics and Child Health, Makerere University, Kampala, Uganda; Medical Research Council Clinical Trials Unit, University College London, United Kingdom; Medical Research Council Clinical Trials Unit, University College London, United Kingdom; Medical Research Council Clinical Trials Unit, University College London, United Kingdom; Infection, Immunity & Inflammation Department, UCL Great Ormond Street Institute of Child Health, London, United Kingdom; Medical Research Council Clinical Trials Unit, University College London, United Kingdom; Paediatric Infectious Diseases Unit, St George's University Hospital, London, United Kingdom; Division of Clinical Pharmacology, Department of Medicine, University of Cape Town, South Africa; Department of Pharmacy, Research Institute for Medical Innovation, Radboud University Medical Center, Nijmegen, The Netherlands; Medical Research Council Clinical Trials Unit, University College London, United Kingdom; Division of Clinical Pharmacology, Department of Medicine, University of Cape Town, South Africa; Wellcome Centre for Infectious Diseases Research in Africa, Institute of Infectious Disease and Molecular Medicine, University of Cape Town, South Africa; Department of Pharmacy, Research Institute for Medical Innovation, Radboud University Medical Center, Nijmegen, The Netherlands

**Keywords:** HIV, TAF, pharmacokinetics, children, drug interaction

## Abstract

**Background:**

We evaluated the pharmacokinetics of tenofovir alafenamide fumarate (TAF) and tenofovir in a subset of African children enrolled in the CHAPAS-4 trial.

**Methods:**

Children aged 3–15 years with human immunodeficiency virus infection failing first-line antiretroviral therapy were randomized to emtricitabine/TAF versus standard-of-care nucleoside reverse transcriptase inhibitor combination, plus dolutegravir, atazanavir/ritonavir, darunavir/ritonavir, or lopinavir/ritonavir. Daily emtricitabine/TAF was dosed according to World Health Organization (WHO)–recommended weight bands: 120/15 mg in children weighing 14 to <25 kg and 200/25 mg in those weighing ≥25 kg. At steady state, 8–9 blood samples were taken to construct pharmacokinetic curves. Geometric mean (GM) area under the concentration–time curve (AUC) and the maximum concentration (C_max_) were calculated for TAF and tenofovir and compared to reference exposures in adults.

**Results:**

Pharmacokinetic results from 104 children taking TAF were analyzed. GM (coefficient of variation [CV%]) TAF AUC_last_ when combined with dolutegravir (n = 18), darunavir/ritonavir (n = 34), or lopinavir/ritonavir (n = 20) were 284.5 (79), 232.0 (61), and 210.2 (98) ng*hour/mL, respectively, and were comparable to adult reference values. When combined with atazanavir/ritonavir (n = 32), TAF AUC_last_ increased to 511.4 (68) ng*hour/mL. For each combination, tenofovir GM (CV%) AUC_tau_ and C_max_ remained below reference values in adults taking 25 mg TAF with a boosted protease inhibitors.

**Conclusions:**

In children, TAF combined with boosted PIs or dolutegravir and dosed according to WHO-recommended weight bands provides TAF and tenofovir concentrations previously demonstrated to be well tolerated and effective in adults. These data provide the first evidence for use of these combinations in African children.

**Clinical Trials Registration:**

ISRCTN22964075.

## Teaser

In a pharmacokinetic substudy of the CHAPAS-4 second-line antiretroviral therapy trial, pharmacokinetic profiles of 104 children showed adequate exposure to tenofovir alafenamide fumarate (TAF) and tenofovir when TAF is dosed according to WHO-recommended weight bands and combined with dolutegravir or ritonavir-boosted protease inhibitors.

Combination antiretroviral therapy (ART) has transformed human immunodeficiency virus (HIV) infection in children from a disease with high morbidity and mortality to a chronic disease. ART including 2 nucleoside/nucleotide reverse trabscriptase inhibitors (NRTI-backbone) and an anchor drug (also referred to as third drug or agent) from a different class is still currently recommended for first- and second-line treatment in most settings. For children, current World Health Organization (WHO) standard-of-care second-line ART options include a backbone of abacavir or zidovudine with lamivudine combined with dolutegravir (DTG), lopinavir/ritonavir (LPV/r), atazanavir/ritonavir (ATV/r), or darunavir/ritonavir (DRV/r) [1]. In adults, the NRTIs emtricitabine (FTC) and tenofovir disoproxil fumarate (TDF) are included as a preferred backbone for second-line ART due to high efficacy in the context of first-line failure, with or without NRTI resistance [2]. However, TDF is generally not recommended in young children due to potential bone and renal toxicity in growing children. The tenofovir prodrug, tenofovir alafenamide fumarate (TAF), achieves 7-fold higher intracellular concentrations of the active metabolite tenofovir diphosphate (TFV-DP) while maintaining lower levels of circulating TFV even with TAF doses about 1/10th of the TDF dose (300 mg for TDF and 25 mg for TAF in adults) [3]. The low dose required for TAF treatment also has the potential to lower the price of ART and allows for a smaller tablet, which is easier for children to take.

In adults, a 10-mg TAF dose combined with the pharmacokinetic booster ritonavir or cobicistat achieves similar exposure compared to 25 mg TAF without a booster [4]. However, 25 mg TAF combined with a boosted protease inhibitor (PI) did not lead to an increase in adverse events [4]. The European Medicines Agency (EMA) and the US Food and Drug Administration (FDA) differ in their dosing recommendations for TAF. The FDA recommends a dose of 25 mg TAF when combined with a boosted antiretroviral regimen while EMA recommends lowering the dose to 10 mg. In the fixed-dose combinations (FDCs) elvitegravir/cobicistat/FTC/TAF and DRV/cobicistat/FTC/TAF, the TAF dose is 10 mg in both the FDA and EMA recommendations [4, 5].

Previous studies on TAF-containing FDCs in children >2 years have reported good viral efficacy with dosing related to both weight and coadministration with ritonavir or cobicistat [6–9]. However, the current guidance on TAF dosing in children mainly relates to the use of FDCs that are not expected to be available in sub-Saharan Africa, a high-HIV-prevalence setting with an urgent need for better, simplified options. With these FDCs, children weighing ≥25 kg receive adult doses: 25 mg and 10 mg TAF in regimens without or with a boosted PI, respectively; for children weighing 14 to <25 kg, 15 mg TAF for an unboosted regimen and 6 mg TAF for a boosted regimen are used.

There are currently no data to support the use of 120/15 mg FTC/TAF and 200/25 mg FTC/TAF formulations in children, in combination with boosted PIs or in combination with the preferred anchor drug DTG.

Here we describe the results of a substudy nested within the CHAPAS-4 (Children with HIV in Africa – Pharmacokinetics and Acceptability of Simple second-line antiretroviral regimens) second-line ART clinical trial, investigating the pharmacokinetics of TAF and TFV when combined with a boosted PI or DTG in children weighing >14 kg.

## METHODS

### Study Design and Participants

CHAPAS-4 (ISRCTN22964075) is an open-label, multicenter, 4 × 2 factorial randomized trial evaluating efficacy and safety of 4 anchor drugs combined with 2 backbone regimens to optimize the second-line treatment of HIV in children aged 3–15 years failing first-line treatment and to better harmonize with adult ART. In this article, we report the pharmacokinetic parameters of TAF and TFV gained from intensive pharmacokinetic substudies nested within the CHAPAS-4 trial. We enrolled children weighing ≥14 kg randomized to FTC/TAF backbone, from 4 sites in Uganda, Zambia, and Zimbabwe. Local ethics committees approved the main trial and pharmacokinetic substudies.

Children were enrolled if their parents/caretakers provided written consent to participate in the CHAPAS-4 trial and the pharmacokinetic substudy; verbal consent was reconfirmed before initiating the pharmacokinetic sampling. Older children provided written assent, as per local country guidelines. The consent and assent documents were translated into local languages. Children weighing 14 to <25 kg received 120/15 mg FTC/TAF; those weighing ≥25 kg received 200/25 mg, both as FDC tablets. FTC/TAF was used in combination with 1 of 4 randomized anchor drugs (ie, DTG, ATV/r, DRV/r, or LPV/r) dosed in weight bands (see Table 1 for drug doses) [1]. We aimed to enroll a minimum of 28 children taking TAF with ATV/r, 18 children taking TAF with DRV/r, and 16 children taking TAF with DTG or LPV/r; more children on ATV/r and DRV/r were included because of the expected extent of the drug interaction with ATV/r and DRV/r.

**Table 1.** Daily Dose of Anchor Drugs taken by CHAPAS-4 participants in World Health Organization–Recommended Weight Bands

Weight Band	DTG	ATV/r	DRV/r	LPV/r
14–19.9 kg	25 mg QD as 5 × 5-mg dispersible tablets	200/75 mg QD (RTV as 3 × 25-mg tablet)	600/100 mg QD	400/100 mg in 2 doses
20–24.9 kg	50 mg QD as film-coated tablet	200/75 mg QD (RTV as 3 × 25-mg tablet)	600/100 mg QD	400/100 mg in 2 doses
25–34.9 kg	50 mg QD as film-coated tablet	300/100 mg QD	800/100 mg QD	600/150 mg in 2 doses 400/100 mg Am and 200/50 mg Pm
≥35 kg	50 mg QD as film-coated tablet	300/100 mg QD	800/100 mg QD	800/200 mg in 2 doses

Abbreviations: ATV/r, atazanavir/ritonavir; DRV/r, darunavir/ritonavir; DTG, dolutegravir; LPV/r, lopinavir/ritonavir; QD, once daily; RTV, ritonavir.

### Procedures

Children with illnesses that could affect pharmacokinetic results, including severe diarrhea, vomiting, renal or liver diseases, and severe malnutrition, and those on concomitant medication known to cause drug–drug interactions with the drugs in the treatment regimen were not eligible. Children were on at least 6 weeks of trial treatment to achieve steady-state plasma concentrations before the 24-hour pharmacokinetic profiles were taken. We took blood samples predose and at 0.5, 1, 2, 4, 6, 8, 12, and 24 hours after observed intake of trial medication with a 250-kcal breakfast. The 0.5-hour sample was added in an amendment to the protocol and was therefore not available for all children. Blood sample volumes were within blood draw limits for children established for research studies [10]. Intake of co-medications other than antiretroviral drugs was not allowed within the first 2 hours after intake of trial medication.

Blood samples were refrigerated within 10 minutes and centrifuged within 1 hour after collection. Plasma was separated and stored at −80°C until shipping to the central laboratory site for quantification. TAF and TFV plasma concentrations were measured at the Division of Clinical Pharmacology, University of Cape Town, South Africa. TAF and TFV were measured simultaneously using a validated and highly sensitive liquid chromatography–tandem mass spectrometry bioanalytical quantification method with a lower limit of quantification of 0.500 ng/mL for both TAF and TFV. The concentration of the analyte found devided by the known concentration of the analyte expressed as a percentage is the accuracy. The combined accuracy of the limit of quantification in low-, medium-, and high-quality controls of TAF and TFV was between 93.8% and 105.1%, with precision (coefficient of variation [%CV]) <13%.

### Noncompartmental Analysis

We considered a pharmacokinetic curve nonevaluable if >1 blood sample was hemolyzed, if protocol deviations had occurred that may have affected the pharmacokinetics of the study drugs (such as use of an interacting concomitant medication), or if a participant was nonadherent based on measured drug concentrations for both the anchor drug and TFV. The criterion for this last exclusion was arbitrarily predefined as the concentration 24 hours after trial medication intake (C_trough_) being >15 times higher than the baseline concentration (C_0_). We used Phoenix 64 software (Pharsight Corporation, Mountain View, California) for noncompartmental analysis (NCA) to determine pharmacokinetic parameters for TAF and TFV. For TFV we report C_trough_, maximum concentration (C_max_), time to maximum plasma concentration (T_max_), and area under the concentration–time curve from dose to 24 hours after dose (AUC_tau_). For TFV we report C_max_, T_max_, and AUC from dose to the time of the last measurable concentration (AUC_last_). AUC was calculated by the linear up/log down trapezoidal method. All samples below the lower limit of quantification (0.500 ng/mL) were omitted. Statistical analysis was performed in R software (version 4.2.2).

For TAF, we compared our observed geometric mean (GM) of AUC_last_ and C_max_ to the GM of the same pharmacokinetic measures in adults taking 25 mg TAF once daily in a boosted or an unboosted regimen [4, 11]. In addition, we report the percentage of children with AUC_last_ >55 ng*hour/mL based on good virological efficacy in adults with this exposure [4, 12].

For TFV, we aimed for the GM AUC_0–24_ to be similar to the AUC_0–24_ in adults taking 25 mg TAF with an unboosted regimen (293 ng*hour/mL), while staying below the AUC observed in adults taking 25 mg TAF combined with a boosted regimen (937 ng*hour/mL) [4, 11]. In addition, we aimed for individual TFV AUC_0–24_ to stay below mean TFV concentrations seen in children taking 8 mg/kg TDF to a maximum of 300 mg (2586 ng*hour/mL), mainly in combination with LPV/r [13]. This exposure to TFV from taking this TDF dose was associated with toxicity in previous pediatric studies [13, 14].

## RESULTS

**Figure 1.**
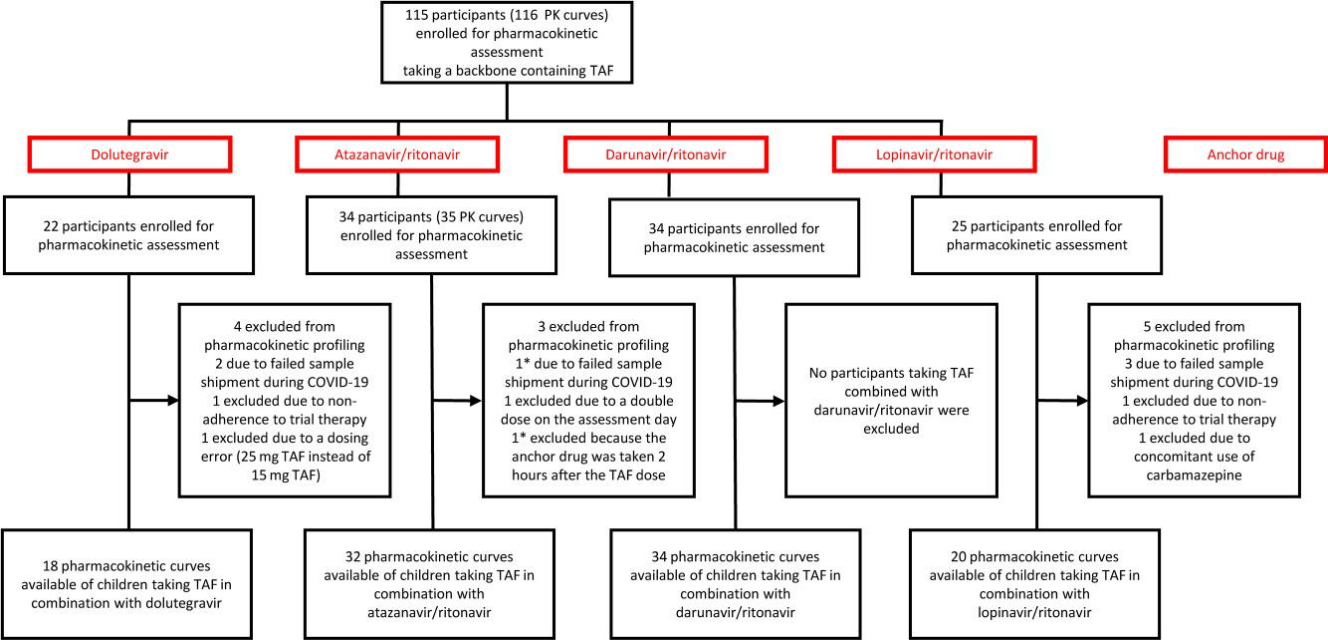
Participant flowchart of children included in the pharmacokinetic substudy. *Two exclusions from the same participant. Abbreviations: COVID-19, Coronavirus Disease 2019; PK, pharmacokinetic; TAF, tenofovir alafenamide fumarate.

Between January 2019 and March 2021, 115 children from Uganda, Zambia, and Zimbabwe randomized to receive a TAF regimen were included in the pharmacokinetic substudy and contributed 116 pharmacokinetic curves. Of the 116 pharmacokinetic curves, 104 curves in 104 children were evaluable and included in the NCA (Figure 1). Twelve pharmacokinetic curves from 11 children were excluded from the NCA due to protocol deviations or shipping issues (Figure 1). Twenty-two of 104 (21%) children did not have a sample at 0.5 hour after dose: 6 on DTG, 6 on ATV/r, 5 on DRV/r, and 5 on LPV/r.

All pharmacokinetic data of TAF and TFV are reported in Table 2. TAF pharmacokinetic results from TAF combined with DTG, DRV/r, or LPV/r were similar to adult reference values (206 ng*hour/mL for adults taking TAF in a regimen without a booster and 222 ng*hour/mL for adults taking 25 mg TAF in a regimen with DRV boosted with cobicistat). TAF combined with ATV/r resulted in significantly higher TAF concentrations than those seen in the other groups: AUC_last_ = 511 (68%) ng*hour/mL; *P* values <.01 (analysis of variance [ANOVA] on log-transformed values with Tukey post hoc analysis). None of the 104 children eligible for NCA had a TAF AUC_last_ <55 ng*hour/mL.

**Table 2.** Demographics of Participants and Pharmacokinetic Parameters of Tenofovir Alafenamide Fumarate and Tenofovir in Combination With 1 of 4 Anchor Drugs

Characteristic	Anchor Drug				Reference Adults	
	TAF + DTG	TAF + ATV/r	TAF + DRV/r	TAF + LPV/r		
TAF dose	<25 kg: 15 mg (n = 9) ≥25 kg: 25 mg (n = 9)	<25 kg: 15 mg (n = 15) ≥25 kg: 25 mg (n = 17)	<25 kg: 15 mg (n = 18) ≥25 kg: 25 mg (n = 16)	<25 kg: 15 mg (n = 8) ≥25 kg: 25 mg (n = 12)	25 mg TAF unboosted/10 mg TAF boosted [4, 11]	25 mg TAF DRV/c [4]
Boosting	Unboosted	Boosted	Boosted	Boosted	Unboosted/boosted	Boosted
No. of participants	18	32	34	20	539	11
Demographics						
Age, y	10.9 (5.46–14.2) [7.64–13.1]	9.98 (4.79–15.0) [6.83–13.2]	10.9 (3.83–14.7) [8.92–12.7]	11.2 (4.27–14.6) [9.38–13.4]	…	…
Weight, kg	25.9 (15.9–53.0) [19.4–35.2]	25.8 (14.5–50.0) [20.4–33.7]	24.0 (14.5–47.0) [21.6–32.7]	25.5 (14.2–48.5) [22.5–41.9]	…	…
BMI, kg/m^2^	16.4 (12.2–21.7) [14.4–17.4]	15.9 (12.8–19.8) [14.4–16.9]	14.8 (12.5–19.0) [13.9–16.5]	15.8 (13.0–20.4) [14.0–18.2]	…	…
Male sex, No. (%)	8 (44%)	15 (47%)	16 (47%)	9 (45%)	…	…
TAF						
AUC_last__(_ng*h/mL)	285 (79)	538 (54)	232 (61)	212 (98)	206 (72)	222 (NR)
C_max_ (ng/mL)	145 (91)	309 (78)	155 (104)	155 (107)	162 (51)	181 (NR)
T_max_	1.1 (0.5–4.0)	2.0 (0.5–6.1)	1.0 (0.5–4.1)	1.0 (0.5–2.0)	…	…
% with AUC >55 ng*h/mL	100%	100%	100%	100%	…	…
TFV						
AUC_tau_, ng*h/mL	324 (29)	847 (37)	744 (26)^a^	864 (46)	293 (27)	937 (NR)
C_max_, ng/mL	19.6 (26)	53.2 (42)	44.5 (26)	50.3 (43)	15.2 (26)	56 (NR)
C_trough_, ng/mL	11.0 (35)	28.1 (39)	26.1 (28)	31.2 (48)	11 (28.5)	33.2 (NR)
T_max_, h	2.0 (1.0–4.0)	3.0 (1.0–6.0)	2.0 (1.0–6.1)	2.0 (0.5–6.0)	…	…
% with AUC <2586 ng*h/mL	100%	100%	100%	100%	…	…

Pharmacokinetic data (except T_max_) are presented as geometric mean with coefficient of variation (CV%); age, weight, BMI, and T_max_ are presented as median (range) [interquartile range].

Abbreviations: ATV/r, atazanavir/ritonavir; AUC, area under the concentration–time curve from 0 to 24 h (AUC_tau_) or from 0 to the last sample with a measurable concentration (AUC_last_); BMI, body mass index; C_max_, highest concentration of pharmacokinetic curve; C_trough_, concentration 24 hours after dose; DRV/c, darunavir/cobicistat; DRV/r, darunavir/ritonavir; DTG, dolutegravir; LPV/r, lopinavir/ritonavir; NR, not reported; TAF, tenofovir alafenamide fumarate; TFV, tenofovir; T_max_, time maximum concentration was reached.

Based on 33 participants because the AUC_tau_ of 1 participant could not be accurately calculated.

GM (CV%) TAF C_max_ in children taking TAF in combination with DTG, DRV/r, and LPV/r was similar to the mean C_max_ observed in adults taking a regimen without a booster. For children taking TAF in combination with ATV/r, the GM (CV%) C_max_ is 86% higher than the C_max_ in adults taking TAF in combination with DRV and cobicistat. The median T_max_ did not vary significantly between anchor drug combination, with only a slight delay observed in combination with ATV/r.

The TFV GM (CV%) AUC_0–24_ of each treatment group stayed between the reference values for adults taking TAF unboosted (293 ng*hour/mL) and boosted (937 ng*hour/mL). The boosted PI arm had significantly higher mean TFV AUC_tau_ than the DTG group (*P* < .01; ANOVA on log-transformed values with Tukey post hoc analysis). However, there were no individuals with TFV AUC_tau_ higher than our predefined maximum reference value of 2586 ng*hour/mL. Furthermore, TFV GM (CV%) C_max_ for each of the treatment groups was between values reported in adults taking TAF with an unboosted regimen (15.2 ng/mL) and adults taking TAF and DRV boosted with cobicistat (56 ng/mL). The median TFV T_max_ was around 2 hours after dose for each treatment group.

TFV C_trough_ was higher than the reference value 11 ng/mL for each group taking TAF with a boosted PI. Children taking TAF with DTG achieved TFV C_trough_ comparable to reference values.

**Figure 2.**
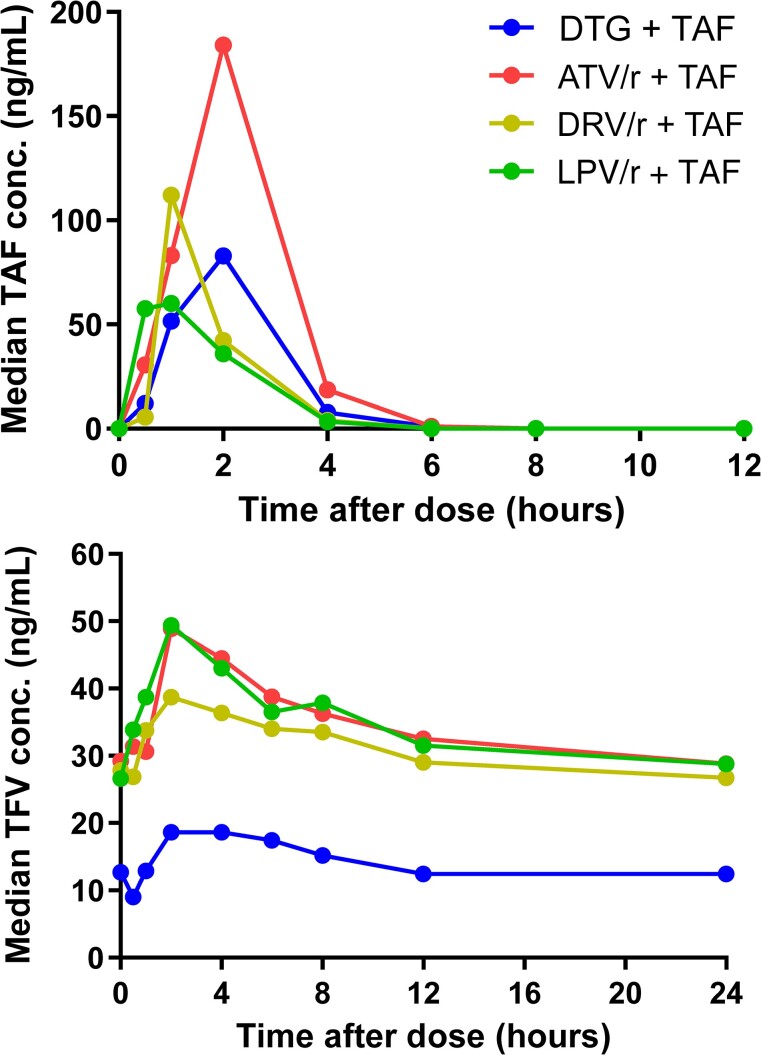
Median tenofovir alafenamide fumarate (TAF) plasma concentration (upper) and mean tenofovir (TFV) plasma concentration (lower) versus time curves of children on TAF combined with dolutegravir (DTG), atazanavir/ritonavir (ATV/r), darunavir/ritonavir (DRV/r), or lopinavir/ritonavir (LPV/r). Twenty-two of 104 (21%) children did not have a sample at 0.5 h after dose: 6 on DTG, 6 on ATV/r, 5 on DRV/r, and 5 on LPV/r.

**Figure 3.**
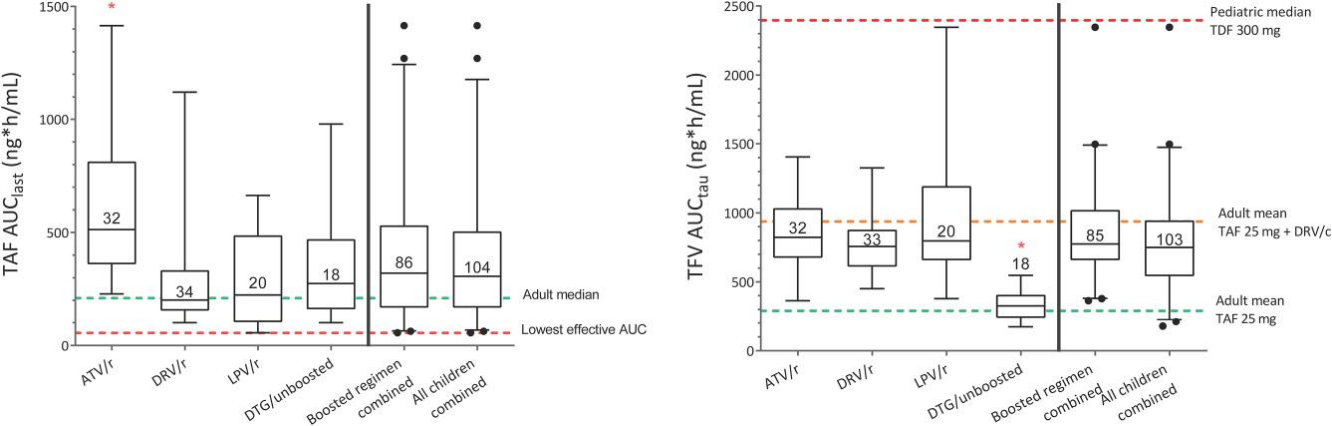
Area under the concentration–time curve (AUC) from 0 to the last sample with a measurable concentration (AUC_last_) of tenofovir alafenamide fumarate (TAF) (left panel) and AUC from 0 to 24 h (AUC_tau_) of tenofovir (right panel) shown with different stratifications of the children in the study: atazanavir/ritonavir, darunavir/ritonavir, lopinavir/ritonavir, dolutegravir, those taking TAF combined with any boosted protease inhibitors (boosted regimen combined), and all children combined. The box and whiskers show median and 2.5th–97.5th percentiles. *Significantly different mean AUC. *P* < .01 (analysis of variance on log-transformed values with Tukey post hoc analysis). Abbreviations: ATV/r, atazanavir/ritonavir; AUC_last_, area under the concentration–time curve from 0 to the last sample; AUC_tau_, area under the concentration–time curve from 0 to 24 hours; DRV/c, darunavir/cobicistat; DRV/r, darunavir/ritonavir; DTG, dolutegravir; LPV/r, lopinavir/ritonavir; TAF, tenofovir alafenamide fumarate; TDF, tenofovir disoproxil fumarate; TFV, tenofovir.

Pharmacokinetic parameters of TAF and TFV by anchor drug combinations are shown in Table 2. Pharmacokinetic profiles of TAF and TFV are shown in Figures 2 and 3.

## DISCUSSION

A dose of 15 mg TAF in children weighing 14 to <25 kg, and 25 mg TAF in children weighing ≥25 kg, in combination with FTC and 1 of 4 anchor drugs achieved GM AUC comparable to target concentrations of TAF and TFV that are safe and effective in adults. No TAF AUC_last_ was observed lower than the predefined efficacy threshold, and no TFV AUC_tau_ was observed higher than the predefined maximum toxicity threshold. Based on a sufficiently similar course of infection and concentration-effect relationship in adults and children, achieving similar exposure parameters in children is generally regarded as a good indicator of drug safety and efficacy and has been used to license drug doses in children [15, 16].

We used TAF AUC_last_ as our primary target for efficacy based on FDA recommendations on TAF dose in children and compared this parameter to adults taking 25 mg TAF in both a boosted and an unboosted antiretroviral regimen. The use of TAF AUC_last_ was also supported by the high viral efficacy that was observed in previous pediatric studies with TAF exposures comparable to adult exposures [6–8, 17]. Moreover, all children in our study achieved a TAF AUC_last_ of at least 55 ng*hour/mL, our minimum exposure target based on adults showing good virological efficacy with this exposure [4, 12].

TFV AUC_tau_ was chosen as the target for safety. This was based on previous FDA guidance of TAF-containing products and previous clinical study results indicating improved safety of TAF versus TDF linked to lower circulating TFV in plasma. TFV plasma concentrations that were achieved in our study were comparable or lower than TFV AUC observed in adults receiving 25 mg TAF in a regimen containing the booster cobicistat. In addition, TFV AUC_tau_ of all individuals (ie, with DTG and with boosted PIs) remained below the median AUC_tau_ observed in children 2 to <12 years old taking the licensed TDF dose of 8 mg/kg with a maximum of 300 mg. The reference value of TFV exposure in children can be considered a conservative target, considering that most children were able to tolerate TDF well, and few children taking TDF with boosted PIs show signs of toxicity [18]. Therefore, we expect results below the median exposure of children taking TDF to be safe.

As expected, and seen in earlier studies in adults, TFV AUC_tau_ and C_max_ concentrations increased when taken with a boosted PI regimen. However, GM concentrations remained below TFV concentrations seen in adults taking DRV boosted with cobicistat. Nonsignificant differences between GM TFV AUC were observed between PI regimens. However, a significant increase was seen in TAF AUC and C_max_ for children combining TAF with ATV/r. The same relative increase was seen in adults combining TAF with ATV/r [19]. Increased TAF exposure when combined with ATV/r could be explained by inhibition of the transporter P-glycoprotein’s active TAF excretion back into the gut and differences in enzyme induction and inhibiting effects attributed to the different PIs [19]. This increase in TAF exposure is not likely to be clinically relevant because TFV levels remain lower than reference values in adults taking TAF with a boosted PI and TFV reference levels of children taking TDF from a study where 92% of children were concomitantly treated with LPV/r.

Our study uses TAF AUC as a proxy for efficacy and TFV AUC as a proxy for safety. It is likely that TFV AUC also correlates to the efficacy of the drug, and likewise, TAF will be indicative of safety. In our study, TAF exposure was significantly higher when it was combined with ATV/r with unknown impact on drug safety. Reassuringly, no safety signal has been raised by the independent data monitoring committee that reviews unblinded trial data regularly. For the other anchor drug combinations, TAF and TFV concentrations were comparable to adult exposure, indicating safe and effective exposure in our study. Tenofovir’s intracellular active metabolite, TFV-DP, could be used as a marker for efficacy. TFV-DP concentrations from dried blood spots as well as sparse pharmacokinetic samples of all children receiving TAF in CHAPAS-4 will be evaluated, and any correlation with viral efficacy and safety will be assessed using pharmacokinetic/pharmacodynamic analysis at the end of the CHAPAS-4 trial.

Safety and efficacy data from the CHAPAS-4 trial are monitored by randomized arm, by the independent data monitoring committee, and will be released at the end of the trial. In particular, renal and bone safety linked to tenofovir use is being assessed by calcaneal ultrasound, dual energy X-ray absorptiometry, and additional biochemistry tests. No safety signal has been reported by the committee.

In adults, the use of TAF has been linked to weight gain, especially when TAF is combined with DTG. Data linking TAF and weight gain are currently not available, but a trial in children receiving DTG has shown no significant increase in body weight with the use of DTG separately [20, 21]. Whether children are also exempt from excessive weight gain when treated with TAF or with a combination of TAF and DTG is currently unknown but will be elucidated when data by randomized arm become available at the end of the CHAPAS-4 trial (expected in early 2024).

A potential limitation of our study is that not all children in our study had a sample taken at 0.5 hours after dose (22 of 104), which could have impacted AUC and C_max_ results. The reason for not including 0.5-hour sample in the original protocol was blood draw volume limits in children. To increase the likelihood of capturing C_max_ of TAF and TFV, a protocol amendment was introduced to include it. This required the pharmacokinetic assessment to be moved to a clinic visit on which fewer blood draws for safety parameters were scheduled. Of the 82 children with a 0.5-hour sample, 21 had reached their maximum TAF concentration at 0.5 hour after dose, and 1 had reached its maximum TFV concentration at 0.5 hours after dose. For this reason, TAF AUC_last_ and C_max_ presented in our results may have been slightly underestimated. However, since TAF AUC_tau_ is our target for efficacy, and there is currently no evidence that the C_max_ is related to toxicity, a higher value would not cause any concern. As only 1 of 83 TFV concentrations reached C_max_ at 0.5 hours, and because TFV has a long half-life, missing C_max_ will have little effect on the total AUC. Therefore, we expect the effect on our safety parameter to be negligible.

TAF has the potential to reduce procurement costs of drug regimens because of the low active dose compared to the current standard-of-care backbone regimens. Furthermore, TAF treatment provides an important alternative to abacavir-containing standard-of-care regimens to children who cannot use TDF due to renal and bone toxicities. Formulations containing TAF have therefore remained in the WHO PADO5 list (Priorities for Antiretroviral Drug Optimization) [22]. This pharmacokinetic substudy bridges the data gap for pharmacokinetic data on TAF combinations in children and provides much-needed evidence on the safe use of TAF in a large cohort of children when given in combination with boosted PIs and DTG. Our pharmacokinetic data suggest that adjusting the TAF dose according to the anchor drug in the combination is not necessary, thus supporting simplification of treatment guidelines. There are also benefits of simplifying drug procurement by national procurement programs.

In conclusion, children aged 3–15 years, weighing ≥14 kg and taking TAF doses according to WHO-recommended weight bands with ritonavir-boosted PIs or DTG, achieved TAF and TFV concentrations that are safe and effective in adults. These data contribute to the practical use of TAF within regimens available in sub-Saharan Africa and other low- and middle-income settings.

## Notes

***Financial support.*** The CHAPAS-4 trial is sponsored by University College London (UCL), with central management by the Medical Research Council (MRC) Clinical Trials Unit at UCL, supported by MRC core funding (Blood Borne Viruses program number: MC_UU_00004/03). The main funding for this study was provided by the European and Developing Countries Clinical Trials Partnership (EDCTP; TRIA2015-1078). Additional funding was received from Janssen Pharmaceuticals and Gilead Sciences. The TAF and TFV assay was developed and performed at the Division of Clinical Pharmacology, University of Cape Town, which is supported by the South African National Institute of Allergy and Infectious Diseases of the South African National Institutes of Health (award numbers UM1 AI068634, UM1 AI068636, and UM1 AI106701). V. Mus. also reports provision of study drugs to institution from ViiV Healthcare, Viatris, and Gilead.

## APPENDIX

### CHAPAS-4 Trial Team Clinical Trials Unit

*Medical Research Council Clinical Trials Unit at University College London:* Di Gibb, Sarah Walker, Anna Turkova, Clare Shakeshaft, Moira Spyer, Margaret Thomason, Anna Griffiths, Lara Monkiewicz, Sue Massingham, Alex Szubert, Alasdair Bamford, Katja Doerholt, Amanda Bigault, Nimisha Dudakia, Annabelle South, Nadine Van Looy, Carly Au, Hannah Sweeney.

### Trial Sites

*Joint Clinical Research Centre Lubowa, Uganda:* Cissy M. Kityo, Victor Musiime, Eva Natukunda, Esether Nambi, Diana Rutebarika Antonia, Rashida Nazzinda, Imelda Namyalo, Joan Nangiya, Lilian Nabeeta, Aidah Nakalyango, Lilian Kobusingye, Caroline Otike, Winnie Namala, Phionah Ampaire, Ayesiga Edgar, Claire Nasaazi, Milly Ndigendawani, Paul Ociti, Priscilla Kyobutungi, Ritah Mbabazi, Phyllis Mwesigwa Rubondo, Juliet Ankunda, Mariam Naabalamba, Mary Nannungi, Alex Musiime, Faith Mbasani, Babu Enoch Louis, Josephine Namusanje, Denis Odoch, Edward Bagirigomwa, Eddie Rubanga, Disan Mulima, Paul Oronon, Eram David Williams, David Baliruno, Josephine Kobusingye, Agnes Uyungrwoth, Barbara Mukanza, Jimmy Okello, Emily Ninsiima, Lutaro Ezra, Christine Nambi, Nansaigi Mangadalen, Musumba Sharif, Nobert B. Serunjogi, Otim Thomas.

*Joint Clinical Research Centre Mbarara, Uganda:* Abbas Lugemwa, Shafic Makumbi, Sharif Musumba, Edward Mawejje, Ibrahim Yawe, Linda Jovia Kyomuhendo, Mariam Kasozi, Rogers Ankunda, Samson kariisa, Christine Inyakuwa, Emily Ninsiima, Lorna Atwine, Beatrice Tumusiime, John Ahuura, Deogracious Tukwasibwe, Violet Nagasha, Judith Kukundakwe, Mariam Zahara Nakisekka, Ritah Winnie Nambejja, Mercy Tukamushaba, Rubinga Baker, Edridah Keminyeto, Barbara Ainebyoona, Sula Myalo, Juliet Acen, Nicholas Jinta Wangwe, Ian Natuhurira, Gershom Kananura Natukunatsa.

*University Teaching Hospital, Zambia:* Veronica Mulenga, Chishala Chabala, Joyce Chipili Lungu, Monica Kapasa, Khonzya Zyambo, Kevin Zimba, Dorothy Zangata, Ellen Shingalili, Naomi Mumba, Nayunda kaonga, Mukumbi Kabesha, Oliver Mwenechanya, Terrence Chipoya, Friday Manakalanga, Stephen Malama, Daniel Chola.

*Arthur Davison Children's Hospital, Zambia:* Bwendo Nduna, Mwate Mwamabazi, Kabwe Banda, Beatrice Kabamba, Muleya Inambao, Pauline Mahy Mukandila, Mwizukanji Nachamba, Stella Himabala, Shadrick Ngosa, Davies Sondashi, Collins Banda, Mark Munyangabe, Grace Mbewe Ngoma, Sarah Chimfwembe, Mercy Lukonde Malasha, Mumba Kajimalwendo, Henry Musukwa, Shadrick Mumba.

*University of Zimbabwe Clinical Research Centre, Zimbabwe:* James Hakim, Mutsa Bwakura-Dangarembizi, Kusum Nathoo, Taneal Kamuzungu, Ennie Chidziva, Joyline Bhiri, Joshua Choga, Hilda Angela Mujuru, Godfrey Musoro, Vivian Mumbiro, Moses Chitsamatanga, Constantine Mutata, Rudo Zimunhu, Shepherd Mudzingwa, Secrecy Gondo, Columbus Moyo, Ruth Nhema, Kathryn Boyd, Farai Matimba, Vinie Kouamou, Richard Matarise, Zorodzai Tangwena, Taona Mudzviti, Allen Matubu, Alfred Kateta, Victor Chinembiri, Dorinda Mukura, Joy Chimanzi, Dorothy Murungu, Wendy Mapfumo, Pia Ngwaru, Lynette Chivere, Prosper Dube, Trust Mukanganiki, Sibusisiwe Weza, Tsitsi Gwenzi, Shirley Mutsai, Misheck Phiri, Makhosonke Ndlovu, Tapiwa Gwaze, Stuart Chitongo, Winisayi Njaravani, Sandra Musarurwa, Cleopatra Langa, Sue Tafeni, Wilbert Ishemunyoro, Nathalie Mudzimirema.

*Mpilo Central Hospital, Zimbabwe:* Wedu Ndebele, Mary Nyathi, Grace Siziba, Getrude Tawodzera, Tracey Makuchete, Takudzwa Chidarura, Shingaidzo Murangandi, Lawrence Mafaro, Owen Chivima, Sifiso Dumani, Beaullar Mampondo, Constance Maphosa, Debra Mwale, Rangarirai Dhlamini, Thabani Sibanda, Nobukhosi Madubeko, Silibaziso Nyathi, Zibusiso Matiwaza.

### Local External Site Monitors

*Uganda:* Sylvia Nabukenya, Harriet Tibakabikoba, Sarah Nakalanzi, Cynthia Williams.

*Zimbabwe:* Precious Chandiwana, Winnie Gozhora, Benedictor Dube.

*Zambia:* Sylvia Mulambo, Hope Mwanyungwi.

### Substudies

*Pharmacokinetic substudies—Radboud University Medical Center:* David Burger, Angela Colbers, Hylke Waalewijn, Lisanne Bevers, Shaghayegh Mohsenian-Naghani.

*Pharmacokinetic substudies—University of Cape Town:* Helen McIlleron, Jennifer Norman, Lubbe Wiesner, Roeland Wasmann, Paolo Denti, Lufina Tsirizani Galileya.

*Toxicity substudy:* Eva Natukunda, Victor Musiime, Phillipa Musoke.

*Health economics substudy—University of York:* Paul Revill, Simon Walker.

### Trial Committees

*Independent Trial Steering Committee Members:* Adeodata Kekitiinwa, Angela Mushavi, Febby Banda Kawamya, Denis Tindyebwa, Hermione Lyall, Ian Weller.

*Independent Data Monitoring Committee Members:* Tim Peto, Philippa Musoke, Margaret Siwale, Rose Kambarami.

### Funders

*European and Developing Countries Clinical Trials Partnership*: Johanna Roth, Pauline Beattie.
